# Structure of Comorbidities and Causes of Death in Patients with Atrial Fibrillation and Chronic Obstructive Pulmonary Disease

**DOI:** 10.3390/jcm14145045

**Published:** 2025-07-16

**Authors:** Stanislav Kotlyarov, Alexander Lyubavin

**Affiliations:** 1 Department of Nursing, Ryazan State Medical University, 390026 Ryazan, Russia; 2 Lipetsk City Hospital №4, 398006 Lipetsk, Russia

**Keywords:** atrial fibrillation, chronic obstructive pulmonary disease, comorbidity, causes of death

## Abstract

**Background/Objectives**: The aim of this study was to assess the structure of comorbidities, the reasons for seeking medical care, and the main causes of fatal outcomes in patients with atrial fibrillation (AF) and chronic obstructive pulmonary disease (COPD). **Methods**: A retrospective analysis of 40,772 electronic medical records in the database of the medical information system with the analysis of medical care requests and causes of fatal outcomes over a 4-year period (from 1 February 2021 to 1 February 2025) was performed. The study participants were divided into three groups. The first group included 1247 participants with AF and COPD (AF + COPD group). The second group included 25,474 patients with AF and without COPD (AF group), and the third group included 14051 patients with COPD and without AF (COPD group). **Results**: Patients with AF + COPD compared to patients with AF alone and COPD alone were more likely to have anemia (5.21% vs. 3.64% and 2.8%, respectively), pulmonary embolism (2.0% vs. 0.52% and 0.46% respectively), type 2 diabetes mellitus (28.2% vs. 22.7% and 14.32%), obesity (24.86% vs. 22.2% and 17.72%), chronic ischemic heart disease (89.25% vs. 78.69% and 49.31%), and chronic heart failure (16.76% vs. 9.47% and 3.22%). In addition, patients with AF + COPD demonstrated the highest mortality among all groups. **Conclusions**: Patients who have both AF and COPD have more comorbidities, seek medical care more frequently, and have worse survival compared with patients with only AF or only COPD.

## 1. Introduction

Chronic obstructive pulmonary disease (COPD) is a widespread, socially significant disease. COPD is a key cause of disability and mortality in many countries and carries a heavy economic and social burden [1,2]. COPD contributes to the development of many other diseases, of which cardiovascular diseases are the most important [3,4,5]. Atherosclerosis in COPD, for example, is an actively studied problem that has shown numerous links between airway inflammation and vascular wall pathobiology. Atrial fibrillation (AF) is another important medical problem that has been associated with a negative impact on quality of life and prognosis. AF is a common arrhythmia among older adults [6,7]. Structural, immune, and electrophysiologic atrial remodeling underlie the development of AF [8,9,10,11]. All these processes are also associated with the resultant effects of inflammation and oxidative stress in COPD [12,13]. It has been established that in AF the number of inflammatory cells in atrial tissues increases [14]. Among these cells, an important place is occupied by macrophages, which participate in the regulation of the electrical activity of cells of the cardiac conduction system. The imbalance in the composition of pro- and anti-inflammatory macrophages is important for the development of myocardial electrical remodeling and AF. Another important pathogenetic mechanism of AF in COPD is chronic hypoxia. AF was more common in patients with declining FEV1 and FEV1/FVC, with declining FEV1 being significantly associated with persistent AF [15]. Atrial fibrosis is the main morphologic substrate for AF development both in patients with and without COPD [16].

Thus, AF and COPD share common pathogenetic mechanisms and, accordingly, are quite common in the same patient, aggravating each other’s course [16]. The prevalence of AF in patients with COPD is about 11–13% [17,18]. But among patients hospitalized with the acute exacerbation of COPD (AE-COPD), the prevalence of AF is higher, with some studies reporting a prevalence of 15–20% [19,20]. Ref. [21] Patients with COPD and AF have higher hospital mortality, longer hospital stays, and increased healthcare costs during COPD exacerbations [22]. A recent study showed that in Asian patients with atrial fibrillation, COPD was found to be associated with an increased annual risk of all-cause mortality and heart failure [23]. Patients with COPD who underwent AF ablation have a higher rate of repeat hospitalizations, although the rate of AF recurrence after ablation is not significantly different from that in patients without COPD [24].

In this regard, various aspects of such comorbidities are of growing interest: from the study of the molecular mechanisms underlying their development to clinical characteristics and life prognosis. Among the clinical aspects of comorbidities, the prevalence of the combination of AF and COPD, as well as concomitant pathology, and the impact of the combination of these diseases on the use of medical care are of interest. These data may be useful in planning the volume of medical care, the identification of new targets to improve the quality of diagnosis, the monitoring of the course, and the selection of optimal therapeutic approaches.

The aim of the study was to assess the structure of comorbidities, the reasons for seeking medical care, and the main causes of fatal outcomes in patients with AF and COPD in the Lipetsk region. The Lipetsk region is a region in the center of the European part of Russia.

## 2. Materials and Methods

**Figure 1:**
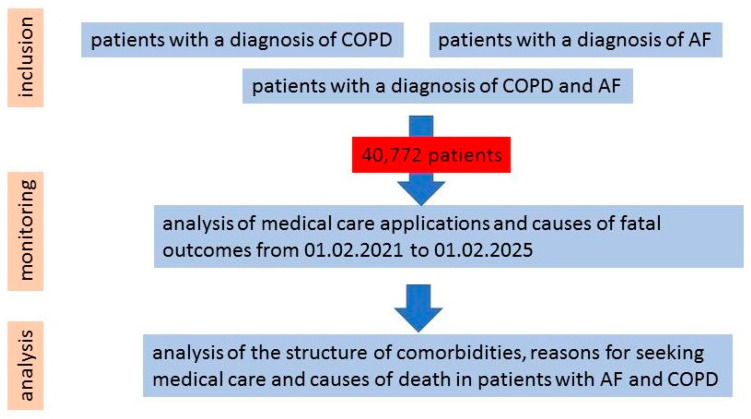
Study design.

This retrospective cohort study is based on the analysis of the anonymized data of patients with COPD and AF who sought medical care in healthcare institutions in the Lipetsk region in the period from 1 February 2021 to 1 February 2025 (4 years) (Figure 1). Anonymized data obtained from the regional medical information system “Kvazar” (Medsoft LLC, https://medsoft.su/services/programmnoe-obespechenie/kmis-kvazar/ (accessed on 3 March 2025)), which contains data on diagnosis, requests for medical care, and causes of death of patients served in medical organizations of the Lipetsk region, were used for the analysis. These data were entered into the medical information system by the physicians from whom the patients sought medical care. Data for analysis were extracted according to the following inclusion and exclusion criteria:

Inclusion criteria:

- Patients older than 18 years of age;
- Confirmed diagnoses of AF and/or COPD, according to current clinical guidelines.

Exclusion criteria:

- Absence of a confirmed diagnosis of AF or COPD.

The sample included data from 40,772 patients who met the inclusion criteria. The following information was collected for each patient: age, sex, comorbidities, dates of seeking medical care, and causes of death, if any. Comorbid conditions were identified based on International Classification of Diseases 10th Revision (ICD-10) codes.

Depending on the presence of COPD and/or AF diagnoses, the patients were divided into three groups. The first group included 1247 patients with COPD and AF (AF + COPD group). The second group included 25,474 patients with AF and without COPD (AF group), and the third group included 14,051 patients with COPD and without AF (COPD group).

The main object of analysis was the structure and prevalence of comorbidities in each of the three groups. Comorbidity was assessed both at the level of nosologic categories (ICD-10 groups) and for individual clinically significant diagnoses. In addition, the reason for seeking medical care (for various diseases) during the study period was analyzed, as well as the distribution of causes of death.

Data analysis and statistical processing were performed using MedCalc by MedCalc Software (https://www.medcalc.org (accessed on 3 March 2025)) and the SciPy library for the Python (version 3.1) programming language (https://scipy.org (accessed on 3 March 2025)). The Shapiro–Wilk test was used to determine the normality of distribution, and the arithmetic mean and standard deviation in M ± SD format were used to evaluate quantitative indicators. To assess the duration of follow-up, the median follow-up was used, with the calculation of the 25th and 75th percentiles in Me [25–75%] format. For categorical variables, differences between groups (including the comparison of the three groups) were compared using the chi-square test. Continuous data were compared between groups as appropriate using Student’s t-test, one-way ANOVA, or the Kruskal–Wallis test (when comparing three groups). When comparing the three groups, Bonferroni correction was applied to assess differences. A value of *p* < 0.05 was considered statistically significant. The logistic regression method was used to analyze the data. To illustrate the outcomes in patients from the analyzed groups, Kaplan–Meier curves were constructed. All-cause mortality, respiratory mortality, and cardiovascular mortality were analyzed. The Kaplan–Meier curves were constructed using the lifelines Python library. The Cox regression model (proportional hazards model) was used to calculate the relative risks of comorbidity influence on the probability of mortality during the follow-up period.

Ethical aspects:

The study was conducted in compliance with ethical standards established by the Declaration of Helsinki, using anonymized data, which excludes the possibility of patient identification. The study was approved by the Ethical Committee of the Ryazan State Medical University (protocol №4 of 9 October 2023).

## 3. Results

The electronic medical records of 40,772 patients with COPD and AF, including 20,013 (49.09%) men and 20,759 (50.91%) women, were retrospectively analyzed. The mean age of the participants was 67.1 ± 16.17 years, with a median age of 70.0 [62.0; 76.0] years.

The prevalence of the combination of AF and COPD in the sample was 4.89% of the total number of patients diagnosed with AF and 8.87% of the total number of patients with COPD. The demographic characteristics of the participants divided into groups are presented in Table 1. Patients in the AF + COPD group were older than patients in the other groups (*p* < 0.01). More males were also recorded in this group compared to in the other groups (*p* < 0.0001). In turn, there were more males among the COPD patients than in the AF group (*p* < 0.0001). It was also found that the COPD patients were significantly younger than the AF patients (*p* < 0.0001).

Analyzing the prevalence of comorbidities, it was found that AF + COPD patients had the highest number of comorbidities for most disease groups (Supplementary Table S1). The analysis of the prevalence of comorbidities in the comparison groups showed that lung cancer was more common in the AF + COPD and COPD groups compared to the AF group (*p* < 0.0001) (Table 2). Anemia was more common in the AF + COPD group compared to the other groups (*p* < 0.01). Hypothyroidism was significantly less common in the COPD group (*p* < 0.001) compared to the AF and AF + COPD groups. The proportion of patients with type 2 diabetes mellitus, obesity, arterial hypertension, angina pectoris, CHD, dilated cardiomyopathy (DCMP), chronic heart failure (CHF), and acute respiratory viral infections was highest in the AF + COPD group and lowest in the COPD group (*p* < 0. 05); however, the proportion of patients with primary myocardial infarction was highest in the AF patients’ group (*p* < 0.05). Also, recurrent myocardial infarction and pulmonary embolism were more common in the AF + COPD patients’ group, and cases of acute viral and bacterial infections were more common in the AF + COPD and COPD patients’ groups (Table 2).

During the 4-year follow-up period, patients in the AF group were significantly less likely to seek medical care for lung cancer compared to patients in the COPD and AF + COPD groups (*p* < 0.0001). The number of visits to medical care facilities for type 2 diabetes mellitus and viral and bacterial pneumonias was highest in the AF + COPD group and lowest in the AF group (*p* < 0.001). The number of visits to medical care facilities for arterial hypertension, chronic CHD, and CVD was maximal in the AF + COPD group and minimal in the COPD group (*p* < 0.0001) (Table 3, Supplemental Table S2).

During the follow-up period, there were 6465 (15.86%) deaths among all participants (Table 4).

The group of patients with a combination of atrial fibrillation and chronic obstructive pulmonary disease (AF + COPD) had the highest mortality among all groups (*p* < 0.0001). When analyzing mortality among participants over 70 years of age, it was found that mortality in the group with isolated atrial fibrillation was statistically significantly lower than in the AF + COPD group (*p* = 0.0076) and also lower than in the group with isolated COPD (*p* < 0.0001) (Table 5).

Patients with COPD more often died from oncologic pathology and nervous system pathology in comparison with the AF group (*p* < 0.01) but not with the AF + COPD group. The number of deaths from cardiovascular disease was highest in the AF + COPD group and lowest in the COPD group (*p* < 0.05). The number of bronchopulmonary deaths was lowest in the AF group (*p* < 0.0001) and did not differ between the AF + COPD and COPD groups. Participants in the AF + COPD group were more likely to die from pulmonary embolism, acute and chronic heart failure, and acute and chronic respiratory failure than those with isolated AF or COPD (Table 6).

**Figure 2:**
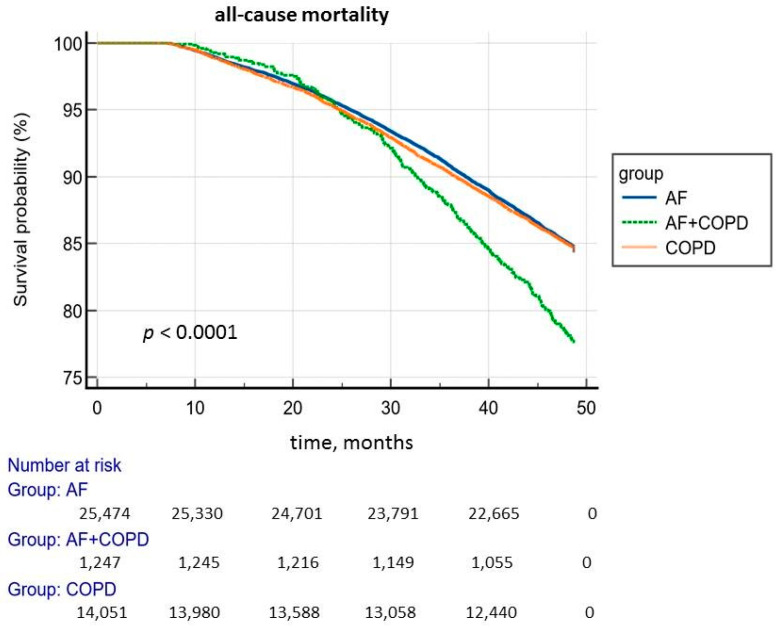
Kaplan–Meier curve for all-cause mortality.

The analysis of the Kaplan–Meier survival curves presented in Figure 2 shows that patients with COPD and AF have worse all-cause mortality compared with the other two patient groups.

**Figure 3:**
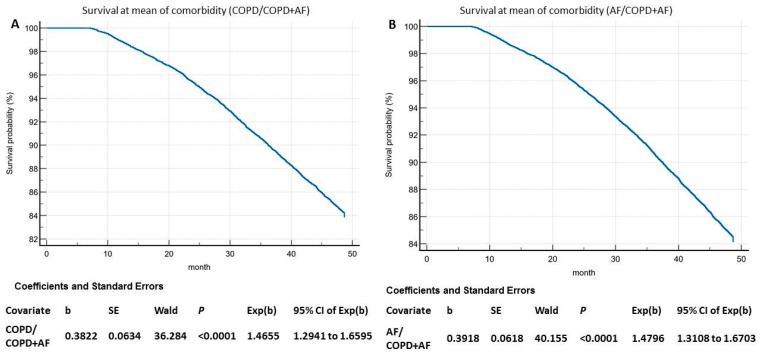
Graph of cumulative survival during the follow-up period analyzed using the Cox regression model. Note: (**A**) Comparison of variables COPD and COPD + AF. (**B**) Comparison of variables AF and COPD + AF.

To assess the influence of comorbidity (COPD/COPD + AF or AF/COPD + AF) on the long-term survival of COPD patients, the four-year survival rates were analyzed using a Cox regression model (Figure 3). The analysis revealed that comorbidity (COPD + AF) significantly predicts an unfavorable survival prognosis for patients with either COPD or AF.

Thus, the group of AF + COPD participants was characterized by the highest mortality from both all causes and from cardiovascular causes but not from respiratory pathology.

## 4. Discussion

The current study analyzed data from a large number of patients with AF and COPD from a medical information system and aimed to assess the prevalence, comorbidity patterns, and impact on the long-term prognosis of the combination of these diseases. This retrospective study included data analysis of a 4-year period (1 February 2021 to 1 February 2025).

The literature data indicate a high prevalence of COPD among patients with AF This is due to the presence of a number of common pathogenetic mechanisms of COPD and AF, which also affect the development of other comorbid diseases. The pathogenetic mechanisms of COPD may influence the major pathways of AF development. Myocardial structural remodeling plays a key role in the pathogenesis of AF and occurs in a shorter time frame than electrical remodeling. It includes changes in the left atrium such as myocyte hypertrophy, myocardial fibrosis, and finally atrial dilation [25]. COPD and pulmonary hypertension significantly affect cardiac hemodynamics and consequently myocardial structural remodeling. In this regard, the study of comorbid diseases associated with COPD is of great practical importance. Patients with COPD have quite a lot of comorbidities. According to Almagro P., the most frequent comorbidities in patients with COPD are arterial hypertension (51.2%), dyslipidemia (36.0%), diabetes mellitus (24.9%), obstructive sleep apnea syndrome (14.9%), heart failure (11.6%), atrial fibrillation (11.5%), peripheral arterial disease (10.4%), and coronary heart disease (10.1%) [26]. According to Vanfleteren LE, more than 97% of patients with COPD have one or more comorbidities, and 53.5% have four or more comorbidities [27].

In our presented study, it was found that patients with COPD are characterized by pronounced comorbidity. The most common comorbidities in this group were arterial hypertension (56.79%), coronary heart disease (CHD) (49.31%), and cerebrovascular disease (CVD) (31.75%). In addition, a significant proportion of patients had metabolic disorders such as diabetes mellitus type 2 (14.32%) and obesity (17.72%). At the same time, participants in the group with a combination of atrial fibrillation and COPD (AF + COPD) demonstrated a significantly higher prevalence of all comorbid conditions studied compared to those in the COPD group without AF. Thus, arterial hypertension (80.35%), CHD (89.25%), CVD (48.2%), diabetes mellitus (28.07%), obesity (24.86%), and angina pectoris (17.24%) were diagnosed more frequently among them. The differences between the groups for all the listed indicators were statistically significant (*p* < 0.05). The higher proportion of several comorbidities in patients in the AF + COPD group is of clinical interest. In the study by Durheim M.T., 16% of patients with AF had COPD. Compared to patients without COPD, patients with COPD were older and more likely to have heart failure and coronary heart disease. Patients with COPD had a higher risk of all-cause mortality (OR 1.52 (95% CI 1.32 to 1.74)) and cardiovascular mortality (OR 1.51 (95% CI 1.24 to 1.84)) [28]. According to Moisés Rodríguez-Mañero, COPD was combined with AF in 11.7% of cases. In the same study, the all-cause mortality was almost twice as high in the AF with COPD group (28.3% vs. 15.5%; *p* < 0.001) [29]. In our study, the all-cause mortality in the AF + COPD participant group was the highest compared to the other two patient groups and was 22.45% (*p* < 0.0001), which is consistent with the reported literature.

In the current study, it was shown that AF + COPD patients were more likely to die from pulmonary embolism than those in the AF or COPD groups. Pulmonary embolism is a known problem in AF, but in the current study, the presence of COPD was shown to further exacerbate this problem in patients with AF. Several studies have shown that the systemic rates of thrombosis are elevated both in stable COPD and during exacerbations. It has been shown, for example, that patients with severe COPD may have an increased risk of secondary venous thromboembolism (VTE) and that patients with COPD and VTE have a higher mortality rate than patients with COPD without VTE [30].

Patients with stable COPD had increased levels of key clotting factors and decreased levels of clotting inhibitors, namely protein S and antithrombin, compared to smokers without COPD. In COPD patients, increased levels of FII, FV, and FX and decreased levels of protein S were observed in patients with more severe disease [31]. At the same time, acute exacerbations of COPD are accompanied by increased plasma fibrinogen and serum IL-6 levels [32]. It should also be noted that the activation and dysfunction of endothelial cells characteristic of COPD are associated not only with inflammation but also with vascular damage with increased coagulation. Increased platelet activation has previously been found in stable COPD and in AE COPD compared with a control group of smokers [33,34,35]. Hypoxia associated with chronic respiratory failure in COPD is also relevant. The HIF-1α-EPO/EDN-1/VEGF pathway has been found to play an important role in the hypercoagulable state in COPD [36]. Thus, the evaluation of coagulation parameters and the prevention of thromboembolism are particularly relevant for patients with AF combined with COPD and AF. In a recent study, COPD was shown to be associated with more frequent use of oral anticoagulants (OACs) [adjusted odds ratio (aOR) and 95% CI: 1.29 (1.13–1.47)] and more frequent discontinuation of OACs [adjusted hazard ratio (aHR) and 95% CI: 1.12 (1.01–1.25)] [37].

The analysis of the number of visits to medical care facilities with different groups of diseases showed that patients with AF + COPD most frequently (24.04 ± 16.98 times during the observation period) sought medical care for diseases of the circulatory system (ICD-10 Code I), with significantly more visits to medical care facilities than patients with COPD alone (9.83 ± 12.3) or with AF alone (19.44 ± 15.36). These data indicate a higher healthcare burden, which is part of the social and economic burden of these diseases. Patients with AF + COPD also had a higher frequency of seeking medical care for diseases of the blood and blood-forming organs and certain disorders involving the immune mechanism (ICD-10 code D). This corresponds to the data obtained in the current study about the higher prevalence of anemia in patients with AF + COPD compared to the other two groups of patients.

Other studies have also shown an association between AF and anemia. One study, for example, showed that the incidence of first-time atrial fibrillation was 9.9% in the general population and 12.8% in a group of patients with anemia [38]. It was also found that the risk of recurrence of paroxysmal atrial fibrillation after catheter ablation was 62.4% in the group with normal blood counts, 78.4% in the group with mild anemia, and 76.2% in the group with moderate-to-severe anemia (*p* < 0.0001) [39]. Anemia is common in patients with COPD, accounting for 7.5% to 33% of patients [40]. Many pathogenetic mechanisms underlie these associations, including systemic inflammation, the dysregulation of iron homeostasis, impaired bone marrow response to erythropoietin, shortened red cell lifespan, comorbidities in COPD, and nutritional disorders contributing to anemia such as renal disease [40]. These data suggest that anemia is an important factor linking the progression of COPD and AF, requiring increased attention to this issue in the management of patients with comorbidities.

The current study also found a higher incidence of endocrine, nutritional, and metabolic diseases (ICD-10 code E). This is consistent with the current study’s findings of a higher prevalence of type 2 diabetes mellitus and obesity in patients with AF + COPD and AF compared to patients with COPD. In another study, diabetes was shown to be associated with an increased risk of atrial fibrillation, and this risk was higher with a longer duration of diabetes and worse glycemic control [41]. In a study in Japanese patients, the prevalence of AF among patients with DM2 was shown to be 5.9%, and it increased with age and was higher in men than in women. In addition, the prevalence of AF increased as albuminuria or proteinuria progressed and the estimated glomerular filtration rate (eGFR) decreased [42]. Overweight and obesity are important risk factors for the development of atrial fibrillation. This association is due to many mechanisms including general hemodynamic changes caused by increased body weight, the effect of excess adipose tissue on systemic inflammation, and oxidative stress, which leads to adverse atrial remodeling [43]. An analysis that included data from 34 studies involving 31,479 patients showed that adipokines, mainly adiponectin, apelin, and resistin, are associated with the risk of developing atrial fibrillation [44,45]. The remodeling that occurs in obesity includes atrial dilation, atrial fibrosis, and consequently altered ionic currents and impaired electrical conduction between atrial myocytes, and this makes atrial tissue more vulnerable to both the occurrence and maintenance of AF [46].

The results of this study showed a higher prevalence of atherosclerotic cardiovascular diseases, including stable angina and PAD, in patients with AF + COPD compared to patients with COPD or AF. It is known that atherosclerotic cardiovascular diseases contribute significantly to the structure of causes of death in patients with COPD [47]. Atherosclerotic cardiovascular diseases share some common pathogenesis links with COPD, based on immune and metabolic mechanisms [48]. Data collected over a 20-year period in Finland showed that coronary heart disease and other vascular diseases were the leading cause of death in patients with COPD (37.3%), followed by COPD itself (30.2%), lung cancer (12.1%), and other malignancies (7.9%) [49]. Reduced FEV1 is itself a risk factor for cardiovascular mortality in COPD patients, regardless of the presence of other known risk factors [50]. Angina is a typical symptom in patients with paroxysmal atrial fibrillation. The high frequency of ventricular contractions, impaired ventricular perfusion, and cardiomyocyte dysfunction in AF may contribute to the development of angina symptoms. AF may accelerate the development of coronary atherosclerosis and increase myocardial oxygen consumption, increasing the mismatch between supply and demand, which contributes to the development or worsening of myocardial ischemia [51]. On the other hand, chronic coronary syndrome alters the structure and function of cardiomyocytes and leads to their replacement by fibrous tissue, which maintains focal ectopic activity in the atrial myocardium [25]. Among COPD patients, the incidence of PAD is as high as 8.8% versus 1.8% among patients without COPD [5,52]. It is believed that COPD and PAD are related through systemic inflammation, the activity of which depends on the frequency of COPD exacerbations.

The results of this study showed a higher prevalence of heart failure in patients with AF + COPD (16.76%) compared to patients with COPD (3.22%) or AF (9.47%). These data are consistent with the pathophysiologic mechanisms linking COPD, AF, and heart failure.

The results of this study confirm the high prevalence of comorbidities among COPD patients. The presence of comorbidities significantly affects the course of COPD, increasing the risk of exacerbations, reducing quality of life, and increasing overall mortality. The obtained data emphasize the need for a comprehensive approach to the management of patients with COPD, including not only the control of respiratory symptoms but also the timely detection and treatment of comorbidities. The observed high frequency of comorbid diseases and requests for medical care in the group of AF + COPD indicates the need for a multidisciplinary approach in the management of these patients.

The current study has some limitations: This study did not analyze the patients’ medication intake, nor did it differentiate between seeking medical help in ambulatory and stationary settings. Also, the current work did not assess the cumulative risks of death from other causes, which is a promising area for future research. The limitations of the study also include the fact that all visits for medical examination and treatment were recorded as cases of seeking medical care, so severe diseases, such as lung cancer, which require more frequent visits for examination and treatment, increase the number of visits per patient. On the other hand, these data suggest an additional burden on the healthcare system for individual diseases. A positive aspect of the current study is that it provides new data on the pattern of comorbidities with which patients sought medical care, which is of considerable practical interest as it may be useful for planning the workload of medical professionals. In addition, information on comorbidities may be useful to modify the management approaches for these patients with comorbidities to improve the diagnosis of the most frequent comorbidities. The advantage of the current study is the large cohort of patients included in the study, which significantly increases the reliability of the data obtained.

Thus, the results of the current study suggest that the combination of COPD and AF is associated with a change in the pattern of comorbidities compared to patients who have these diseases separately. The presence of atrial fibrillation in patients with COPD is associated with a significantly higher comorbid background. This emphasizes the need for more careful clinical monitoring and a multidisciplinary approach to the management of this category of patients.

## 5. Conclusions

The results of our study showed that patients in the group with a combination of AF and COPD demonstrate significant comorbidity, manifested in a wide range of comorbidities affecting various organs and systems. These patients are characterized by a higher frequency of seeking medical care compared to groups with isolated diagnoses, indicating a more severe clinical picture and a high level of medical needs. Given the high comorbidity and frequency of referrals, the combination of AF and COPD requires special attention in clinical practice, as it can significantly worsen the prognosis and quality of life of patients, increasing the need for a comprehensive approach to treatment and monitoring. Understanding the clinical significance of comorbidity in the combination of AF and COPD is important for optimizing treatment strategies and the timely detection and treatment of comorbidities, as well as for improving the patients’ quality of life and survival.

## Figures and Tables

**Table 1:** Demographic characteristics of the groups.

Parameter	AF + COPD (*n* = 1247)	AF (*n* = 25,474)	COPD (*n* = 14,051)	Significance Level, *p* *
	1	2	3	
Age, years	71.82 ± 9.31	70.8 ± 10.8	59.97 ± 21.48	*p* < 0.0001 *p* ^1,2^ = 0.0011 *p* ^2,3^ < 0.0001 *p* ^1,3^ < 0.0001
Male	773 (61.99%)	11,243 (44.14%)	7997 (56.91%)	-
Female	474 (38.01%)	14,231 (55.86%)	6054 (43.09%)	-

* Superscripts ^1,2,3^ denote the numbers of the groups being compared.

**Table 2:** Prevalence of individual clinical conditions.

Clinical Condition	AF + COPD (*n* = 1247)	AF (*n* = 25,474)	COPD (*n* = 14,051)	Significance Level, *p* *	Odds Ratio (OR) and 95% Confidence Interval (CI) *
	1	2	3		
Lung cancer	58 (4.65%)	254 (1.0%)	543 (3.86%)	*p* < 0.001 *p* ^1,2^ < 0.0001 *p* ^2,3^ < 0.0001 *p* ^1,3^ = 0.1955	OR ^1,2^ 4.84 95% CI (3.55–6.51) OR ^2,3^ 0.25 95% CI (0.21–0.29) OR ^1,3^ 1.21 95% CI (0.9–1.6)
Anemia	65 (5.21%)	927 (3.64%)	393 (2.8%)	*p* < 0.0001 *p* ^1,2^ = 0.0052 *p* ^2,3^=0.0001 *p* ^1,3^ < 0.0001	OR ^1,2^ 1.45 95% CI (1.11–1.89) OR ^2,3^ 1.31 95% CI (1.16–1.48) OR ^1,3^ 1.91 95% CI (1.44–2.51)
Hypothyroidism	103 (8.26%)	2266 (8.9%)	788 (5.61%)	*p* < 0.0001 *p* ^1,2^ = 0.4716 *p* ^2,3^ < 0.0001 *p* ^1,3^ = 0.0002	OR ^1,2^ 1.11 95% CI (0.82–1.48) OR ^2,3^ 1.69 95% CI (1.49–1.93) OR ^1,3^ 1.88 95% CI (1.38–2.55)
Type 1 DM	53 (4.25%)	976 (3.83%)	323 (2.3%)	*p* < 0.001 *p* ^1,2^ = 0.4996 *p* ^2,3^ < 0.0001 *p* ^1,3^ < 0.0001	OR ^1,2^ 1.11 95% CI (0.82–1.48) OR ^2,3^ 1.69 95% CI (1.49–1.93) OR ^1,3^ 1.88 95% CI (1.38–2.55)
Type 2 DM	350 (28.07%)	5782 (22.7%)	2012 (14.32%)	*p* < 0.0001 *p* ^1,2^ < 0.0001 *p* ^2,3^ < 0.0001 *p* ^1,3^ < 0.0001	OR ^1,2^ 1.32 95% CI (1.17–1.51) OR ^2,3^ 1.75 95% CI (1.66–1.86) OR ^1,3^ 2.33 95% CI (2.04–2.67)
Obesity	310 (24.86%)	5654 (22.2%)	2490 (17.72%)	*p* < 0.0001 *p* ^1,2^ = 0.0299 *p* ^2,3^ < 0.0001 *p* ^1,3^ < 0.0001	OR ^1,2^ 1.15 95% CI (1.01–1.32) OR ^2,3^ 1.32 95% CI (1.26–1.4) OR ^1,3^ 1.53 95% CI (1.34–1.76)
AH	1002 (80.35%)	19419 (76.23%)	7980 (56.79%)	*p* < 0.0001 *p* ^1,2^ = 0.0009 *p* ^2,3^ < 0.0001 *p* ^1,3^ < 0.0001	OR ^1,2^ 1.27 95% CI (1.1–1.48) OR ^2,3^ 2.43 95% CI (2.33–2.55) OR ^1,3^ 3.11 95% CI (2.69–3.61)
Angina pectoris	215 (17.24%)	3085 (12.11%)	911 (6.48%)	*p* < 0.0001 *p* ^1,2^ < 0.0001 *p* ^2,3^ < 0.0001 *p* ^1,3^ < 0.0001	OR ^1,2^ 1.51 95% CI (1.29–1.76) OR ^2,3^ 1.98 95% CI (1.84–2.15) OR ^1,3^ 3.00 95% CI (2.54–3.54)
Primary MI	19 (1.52%)	651 (2.56%)	185 (1.32%)	*p* < 0.0001 *p* ^1,2^ = 0.029 *p* ^2,3^ < 0.0001 *p* ^1,3^ = 0.5413	OR ^1,2^ 0.58 95% CI (0.35–0.93) OR ^2,3^ 1.96 95% CI (1.66–2.33) OR ^1,3^ 1.15 95% CI (0.68–1.87)
Recurrent MI	8 (0.64%)	68 (0.27%)	23 (0.16%)	*p* ^1,2^ = 0.0313 *p* ^2,3^ = 0.0523 *p* ^1,3^ = 0.0011	OR ^1,2^ 2.41 95% CI (1.0–5.04) OR ^2,3^ 1.63 95% CI (1.0–2.75) OR ^1,3^ 3.93 95% CI (1.52–9.14)
CHD	1113 (89.25%)	20045 (78.69%)	6929 (49.31%)	*p* < 0.0001 *p* ^1,2^ < 0.0001 *p* ^2,3^ < 0.0001 *p* ^1,3^ < 0.0001	OR ^1,2^ 2.24 95% CI (1.87–2.72) OR ^2,3^ 3.79 95% CI (3.63–3.97) OR ^1,3^ 8.53 95% CI (7.11–10.32)
PE	25 (2.0%)	133 (0.52%)	65 (0.46%)	*p* < 0.0001 *p* ^1,2^ < 0.0001 *p* ^2,3^ = 0.4669 *p* ^1,3^ < 0.0001	OR ^1,2^ 3.89 95% CI (2.43–6.04) OR ^2,3^ 1.12 95% CI (0.83–1.54) OR ^1,3^ 4.40 95% CI (2.65–7.11)
Aortic stenosis	13 (1.04%)	257 (1.01%)	50 (0.36%)	*p* < 0.0001 *p* ^1,2^ = 0.90 *p* ^2,3^ < 0.0001 *p* ^1,3^ = 0.0007	OR ^1,2^ 1.03 95% CI (0.54–1.81) OR ^2,3^ 2.85 95% CI (2.1–3.95) OR ^1,3^ 2.94 95% CI (1.47–5.53)
DCMP	49 (3.93%)	633 (2.48%)	142 (1.01%)	*p* < 0.0001 *p* ^1,2^ = 0.0022 *p* ^2,3^ < 0.0001 *p* ^1,3^ < 0.0001	OR ^1,2^ 1.60 95% CI (1.17–2.16) OR ^2,3^ 2.49 95% CI (2.07–3.02) OR ^1,3^ 4.00 95% CI (2.82–5.61)
CHF	209 (16.76%)	2412 (9.47%)	453 (3.22%)	*p* < 0.0001 *p* ^1,2^ < 0.0001 *p* ^2,3^ < 0.0001 *p* ^1,3^ < 0.0001	OR ^1,2^ 1.92 95% CI (1.64–2.25) OR ^2,3^ 3.13 95% CI (2.83–3.49) OR ^1,3^ 6.04 95% CI (5.04–7.22)
ICH	4 (0.32%)	149 (0.58%)	38 (0.27%)	*p* < 0.0001 *p* ^1,2^ = 0.3102 *p* ^2,3^ < 0.0001 *p* ^1,3^ = 0.7447	OR ^1,2^ 0.54 95% CI (0.15–1.43) OR ^2,3^ 2.16 95% CI (1.51–3.19) OR ^1,3^ 1.18 95% CI (0.31–3.3)
IS	112 (8.98%)	2355 (9.24%)	438 (3.12%)	*p* < 0.0001 *p* ^1,2^ = 0.7539 *p* ^2,3^ < 0.0001 *p* ^1,3^ < 0.0001	OR ^1,2^ 0.96 95% CI (0.79–1.18) OR ^2,3^ 3.16 95% CI (2.85–3.52) OR ^1,3^ 3.06 95% CI (2.45–3.82)
CVD	601 (48.2%)	10311 (40.48%)	4461 (31.75%)	*p* < 0.0001 *p* ^1,2^ < 0.0001 *p* ^2,3^ < 0.0001 *p* ^1,3^ < 0.0001	OR ^1,2^ 1.36 95% CI (1.22–1.54) OR ^2,3^ 1.46 95% CI (1.4–1.53) OR ^1,3^ 1.99 95% CI (1.78–2.25)
PAD	13 (1.04%)	102 (0.4%)	58 (0.41%)	*p* = 0.0029 *p* ^1,2^ = 0.0016 *p* ^2,3^ = 0.9182 *p* ^1,3^ = 0.0035	OR ^1,2^ 2.62 95% CI (1.35–4.7) OR ^2,3^ 0.96 95% CI (0.7–1.36) OR ^1,3^ 2.54 95% CI (1.27–4.7)
ARVI	588 (47.15%)	10079 (39.57%)	7227 (51.43%)	*p* < 0.0001 *p* ^1,2^ < 0.0001 *p* ^2,3^ < 0.0001 *p* ^1,3^ = 0.0041	OR ^1,2^ 1.36 95% CI (1.21–1.53) OR ^2,3^ 0.61 95% CI (0.59–0.64) OR ^1,3^ 0.84 95% CI (0.75–0.95)
Viral pneumonia	180 (14.43%)	2030 (7.97%)	1504 (10.7%)	*p* < 0.0001 *p* ^1,2^ < 0.0001 *p* ^2,3^ < 0.0001 *p* ^1,3^ < 0.0001	OR ^1,2^ 1.94 95% CI (1.64–2.3) OR ^2,3^ 0.72 95% CI (0.67–0.78) OR ^1,3^ 1.40 95% CI (1.18–1.67)
Bacterial pneumonia	188 (15.08%)	1787 (7.01%)	1615 (11.49%)	*p* < 0.0001 *p* ^1,2^ < 0.0001 *p* ^2,3^ < 0.0001 *p* ^1,3^ = 0.0002	OR ^1,2^ 2.35 95% CI (1.99–2.77) OR ^2,3^ 0.58 95% CI (0.54–0.62) OR ^1,3^ 1.36 95% CI (1.15–1.61)

Note: ICH—intracerebral hemorrhage; DCMP—dilated cardiomyopathy; AH—hypertension; IS—ischemic stroke; MI—myocardial infarction; PAD—peripheral artery disease; ARVI—acute respiratory viral infection; DM—diabetes mellitus; PE—pulmonary embolism; CHD—chronic ischemic heart disease; CHF—chronic heart failure; CVD—cerebrovascular disease. * Superscripts ^1,2,3^ denote the numbers of the groups being compared.

**Table 3:** Number of requests for medical care during the 4-year observation period.

Clinical Condition	AF + COPD (*n* = 1247)	AF (*n* = 25,474)	COPD (*n* = 14,051)	Significance Level, *p* *
	1	2	3	
Lung cancer	0.54 ± 3.35	0.13 ± 1.83	0.55 ± 3.87	*p* < 0.001 *p* ^1,2^ < 0.0001 *p* ^2,3^ < 0.0001 *p* ^1,3^ = 0.9311
Anemia	0.1 ± 0.59	0.07 ± 0.53	0.06 ± 0.49	*p* < 0.001 *p* ^1,2^ = 0.1014 *p* ^2,3^ = 0.0014 *p* ^1,3^ = 0.0035
Hypothyroidism	0.19 ± 0.85	0.26 ± 1.14	0.15 ± 0.87	*p* < 0.001 *p* ^1,2^ = 0.0405 *p* ^2,3^ < 0.0001 *p* ^1,3^ = 0.1623
Type 1 DM	0.2 ± 1.72	0.19 ± 1.83	0.14 ± 1.56	*p* < 0.001 *p* ^1,2^ = 0.8544 *p* ^2,3^ = 0.0031 *p* ^1,3^ = 0.1693
Type 2 DM	2.8 ± 6.93	2.24 ± 6.5	1.41 ± 5.18	*p* < 0.001 *p* ^1,2^ = 0.0032 *p* ^2,3^ < 0.0001 *p* ^1,3^ < 0.0001
Obesity	0.37 ± 0.75	0.34 ± 0.76	0.29 ± 0.77	*p* < 0.001 *p* ^1,2^ = 0.1603 *p* ^2,3^ < 0.0001 *p* ^1,3^ = 0.0004
AH	5.76 ± 6.42	4.98 ± 5.64	3.68 ± 5.37	*p* < 0.0001 *p* ^1,2^ < 0.0001 *p* ^2,3^ < 0.0001 *p* ^1,3^ < 0.0001
Angina pectoris	0.61 ± 2.18	0.41 ± 1.89	0.25 ± 1.57	*p* < 0.0001 *p* ^1,2^ = 0.0003 *p* ^2,3^ < 0.0001 *p* ^1,3^ < 0.0001
Primary MI	0.03 ± 0.34	0.05 ± 0.38	0.03 ± 0.28	*p* < 0.001 *p* ^1,2^ = 0.1737 *p* ^2,3^ < 0.0001 *p* ^1,3^ = 0.3039
Recurrent MI	0.01 ± 0.11	0.0 ± 0.12	0.0 ± 0.05	*p* < 0.001 *p* ^1,2^ = 0.338 *p* ^2,3^ = 0.0058 *p* ^1,3^ = 0.0006
CHD	7.4 ± 7.29	5.52 ± 6.45	3.07 ± 5.32	*p* < 0.0001 *p* ^1,2^ < 0.0001 *p* ^2,3^ < 0.0001 *p* ^1,3^ < 0.0001
PE	0.04 ± 0.43	0.01 ± 0.17	0.01 ± 0.2	*p* < 0.0001 *p* ^1,2^ < 0.0001 *p* ^2,3^ = 0.6958 *p* ^1,3^ < 0.0001
Aortic stenosis	0.04 ± 0.69	0.05 ± 0.77	0.02 ± 0.42	*p* < 0.001 *p* ^1,2^ = 0.8218 *p* ^2,3^ < 0.0001 *p* ^1,3^ = 0.0345
DCMP	0.25 ± 1.78	0.12 ± 1.28	0.05 ± 0.77	*p* < 0.0001 *p* ^1,2^ = 0.0007 *p* ^2,3^ < 0.0001 *p* ^1,3^ < 0.0001
CHF	0.43 ± 1.3	0.23 ± 0.96	0.07 ± 0.55	*p* < 0.0001 *p* ^1,2^ < 0.0001 *p* ^2,3^ < 0.0001 *p* ^1,3^ < 0.0001
ICH	0.0 ± 0.09	0.01 ± 0.16	0.0 ± 0.1	*p* < 0.001 *p* ^1,2^ = 0.2998 *p* ^2,3^ = 0.0005 *p* ^1,3^ = 0.8751
IS	0.21 ± 0.92	0.21 ± 0.91	0.06 ± 0.48	*p* < 0.0001 *p* ^1,2^ = 0.8041 *p* ^2,3^ < 0.0001 *p* ^1,3^ < 0.0001
CVD	1.67 ± 2.86	1.37 ± 2.83	1.07 ± 2.56	*p* < 0.0001 *p* ^1,2^ = 0.0002 *p* ^2,3^ < 0.0001 *p* ^1,3^ < 0.0001
PAD	0.06 ± 0.72	0.01 ± 0.3	0.02 ± 0.35	*p* < 0.0001 *p* ^1,2^ < 0.0001 *p* ^2,3^ = 0.4396 *p* ^1,3^ = 0.0005
ARVI	0.11 ± 0.39	0.1 ± 0.4	0.15 ± 0.53	*p* < 0.0001 *p* ^1,2^ = 0.5533 *p* ^2,3^ < 0.0001 *p* ^1,3^ = 0.0085
Viral pneumonia	0.26 ± 0.79	0.12 ± 0.48	0.17 ± 0.61	*p* < 0.0001 *p* ^1,2^ < 0.0001 *p* ^2,3^ < 0.0001 *p* ^1,3^ < 0.0001
Bacterial pneumonia	0.25 ± 0.8	0.1 ± 0.42	0.19 ± 0.65	*p* < 0.0001 *p* ^1,2^ < 0.0001 *p* ^2,3^ < 0.0001 *p* ^1,3^ = 0.0043

Note: ICH—intracerebral hemorrhage; DCMP—dilated cardiomyopathy; AH—arterial hypertension; IS—ischemic stroke; MI—myocardial infarction; PAD—peripheral artery disease; ARVI—acute respiratory viral infection; DM—diabetes mellitus; PE—pulmonary embolism; CHD—chronic ischemic heart disease; CHF—chronic heart failure; CVD—cerebrovascular disease. * Superscripts ^1,2,3^ denote the numbers of the groups being compared.

**Table 4:** Characteristics of deceased participants by sex and age.

Parameter	AF + COPD (*n* = 1247)	AF (*n* = 25,474)	COPD (*n* = 14,051)	Significance Level, *p* *	Hazard Ratio (HR) and 95% Confidence Interval (CI) *
	1	2	3		
Male	206 (73.57%)	1967 (49.42%)	1655 (75.06%)	*p* < 0.0001 *p* ^1,2^ < 0.0001 *p* ^2,3^ < 0.0001 *p* ^1,3^ = 0.6407	HR ^1,2^ 1.58 95% CI (1.37–1.82) HR ^2,3^ 1.20 95% CI (1.13–1.28)
Female	74 (26.43%)	2013 (50.58%)	550 (24.94%)	*p* < 0.0001 *p* ^1,2^ < 0.0001 *p* ^2,3^ < 0.0001 *p* ^1,3^ = 0.6407	HR^2,3^ 0.62 95% CI (0.57–0.68)
Age	73.46 ± 9.94	75.69 ± 10.54	72.89 ± 11.37	*p* < 0.0001 *p* ^1,2^ = 0.0006 *p* ^2,3^ < 0.0001 *p* ^1,3^ = 0.4265	-

Note: * Superscripts ^1,2,3^ denote the numbers of the groups being compared.

**Table 5:** Mortality rate in the groups.

Parameter	AF + COPD (*n* = 1247)	AF (*n* = 25,474)	COPD (*n* = 14,051)	Significance Level, *p* *	Hazard Ratio (HR) and 95% Confidence Interval (CI) *
	1	2	3		
Total number of deceased	280 (22.45%)	3980 (15.62%)	2205 (15.69%)	*p* < 0.0001 *p* ^1,2^ < 0.0001 *p* ^2,3^ = 0.8678 *p* ^1,3^ < 0.0001	HR ^1,2^ 1.47 95% CI (1.31–1.67) HR ^1,3^ 1.46 95% CI (1.29–1.65)
Over 70 years old	176 (24.89%)	2800 (20.64%)	1279 (27.19%)	*p* < 0.0001 *p* ^1,2^ = 0.0076 *p* ^2,3^ < 0.0001 *p* ^1,3^ = 0.2157	HR ^1,2^ 1.26 95% CI (1.09–1.47) HR ^2,3^ 1.38 95% CI (1.29–1.47)

Note: * Superscripts ^1,2,3^ denote the numbers of the groups being compared.

**Table 6:** Characterization of study participants by cause of death (ICD-10 groups).

Diseases Group (ICD-10)	AF + COPD (*n* = 1247)	AF (*n* = 25,474)	COPD (*n* = 14,051)	Significance Level, *p* *	Hazard Ratio (HR) and 95% Confidence Interval (CI) *
	1	2	3		
Neoplasms	13 (1.04%)	167 (0.66%)	132 (0.94%)	*p* = 0.0043 *p* ^1,2^ = 0.146 *p* ^2,3^ = 0.0022 *p* ^1,3^ = 0.7189	HR ^2,3^ 1.26 95% CI (1.05–1.52)
Diseases of the nervous system	28 (2.25%)	737 (2.89%)	215 (1.53%)	*p* < 0.0001 *p* ^1,2^ = 0.2105 *p* ^2,3^ < 0.0001 *p* ^1,3^ = 0.0691	HR ^2,3^ 0.59 95% CI (0.52–0.68)
Diseases of the circulatory system	171 (13.71%)	2127 (8.35%)	1070 (7.62%)	*p* < 0.0001 *p* ^1,2^ < 0.0001 *p* ^2,3^ = 0.0109 *p* ^1,3^ < 0.0001	HR ^1,2^ 1.64 95% CI (1.41–1.92) HR ^2,3^ 0.92 95% CI (0.85–0.99) HR ^1,3^ 1.78 95% CI (1.52–2.09)
Pulmonary embolism	15 (1.20%)	109 (0.43%)	58 (0.41%)	*p* = 0.0258 *p* ^1,2^ = 0.3125 *p* ^2,3^ = 0.0716 *p* ^1,3^ = 0.0388	HR ^1,3^ 1.70 95% CI (1.02–2.83)
I50.0 congestive heart failure	53 (4.25%)	941 (3.69%)	249 (1.77%)	*p* < 0.0001 *p* ^1,2^ = 0.3489 *p* ^2,3^ < 0.0001 *p* ^1,3^ < 0.0001	HR ^2,3^ 0.54 95% CI (0.47–0.61) HR ^1,3^ 2.12 95% CI (1.59–2.83)
I50.1 left ventricular failure	36 (2.89%)	694 (2.72%)	267 (1.9%)	*p* < 0.0001 *p* ^1,2^ = 0.7988 *p* ^2,3^ < 0.0001 *p* ^1,3^ = 0.022	HR ^2,3^ 0.74 95% CI (0.65–0.84) HR ^1,3^ 1.39 95% CI (1.00–1.95)
Diseases of the respiratory system	30 (2.41%)	198 (0.78%)	375 (2.67%)	*p* < 0.0001 *p* ^1,2^ < 0.0001 *p* ^2,3^ < 0.0001 *p* ^1,3^ = 0.6437	HR ^1,2^ 2.27 95% CI (1.57–3.26) HR ^2,3^ 2.53 95% CI (2.19–2.93)
J96.0 acute respiratory failure	12 (0.96%)	127 (0.5%)	172 (1.22%)	*p* < 0.0001 *p* ^1,2^ = 0.0433 *p* ^2,3^ < 0.0001 *p* ^1,3^ = 0.4982	HR ^2,3^ 1.77 95% CI (1.48–2.12)
J96.1 chronic respiratory failure	14 (1.12%)	28 (0.11%)	145 (1.03%)	*p* < 0.0001 *p* ^1,2^ < 0.0001 *p* ^2,3^ < 0.0001 *p* ^1,3^ = 0.8751	HR ^1,2^ 2.52 95% CI (1.50–4.23) HR ^2,3^ 2.68 95% CI (2.16–3.32)

Note: * Superscripts ^1,2,3^ denote the numbers of the groups being compared.

## Data Availability

The raw data supporting the conclusions of this article will be made available by the authors on request.

## Supplementary Materials

The following supporting information can be downloaded at: https://www.mdpi.com/article/10.3390/jcm14145045/s1, Table S1: Prevalence of comorbidities by ICD-10 groups; Table S2: Number of visits to medical care during the observation period with diseases from different ICD-10 groups.

## Abbreviations

The following abbreviations are used in this manuscript:

AF	Atrial fibrillation
AH	Arterial hypertension
ARVI	Acute respiratory viral infection
CHD	Chronic ischemic heart disease
CHF	Chronic heart failure
COPD	Chronic obstructive pulmonary disease
CVD	Cerebrovascular disease
DCMP	Dilated cardiomyopathy
DM	Diabetes mellitus
ICD	International Classification of Diseases
ICH	Intracerebral hemorrhage
IS	Ischemic stroke
MI	Myocardial infarction
PAD	Peripheral artery disease
PE	Pulmonary embolism
