# Supplementary material for: Structure of Comorbidities and Causes of Death in Patients with Atrial Fibrillation and Chronic Obstructive Pulmonary Disease
Source: J Clin Med. 2025 Jul 16;14(14):5045. doi: 10.3390/jcm14145045 (PMC12295669; doi:10.3390/jcm14145045)
Supplement: Supplementary file 1 [file jcm-14-05045-s001.zip › jcm-3714050-supplementary.pdf]

Supplemental Table S1. Prevalence of comorbidities by ICD-10 groups.

ICD-10 group (first character of code)	AF+COPD (*n*=1,247)	AF (*n*=25,474)	COPD (*n*=14,051)	Significance level, *p**	Odds ratio (OR) and 95% confidence interval (CI)*
	1	2	3		
A	50 (4.01%)	516 (2.03%)	397 (2.83%)	p<0.0001 $p^{1,2}$<0.0001 $p^{2,3}$<0.0001 $p^{1,3}$=0.0219	OR[1,2] 2.02 95% CI (1.47-2.72) OR [2,3] 0.71 95% CI (0.62-0.81) OR[1,3] 1.43 95% ДИ (1.04-1.94)
B	114 (9.14%)	1761 (6.91%)	1650 (11.74%)	p<0.0001 $p^{1,2}$=0.0032 $p^{2,3}$<0.0001 $p^{1,3}$=0.0067	OR[1,2] 1.35 95% CI (1.1-1.65) OR[2,3] 0.55 95% ДИ (0.52-0.6) OR[1,3] 0.75 95% ДИ (0.61-0.92)
C	216 (17.32%)	3504 (13.76%)	1833 (13.05%)	p= 0,0001 $p^{1,2}$=0.0004 $p^{2,3}$=0.0498 $p^{1,3}$=0.0067	OR[1,2] 1.31 95% CI (1.12-1.53) OR[2,3] 1.06 95% CI (1.0-1.13) OR[1,3] 1.39 95% CI (1.19-1.63)
D	422 (33.84%)	6273 (24.63%)	3626 (25.81%)	p<0.0001 $p^{1,2}$<0.0001 $p^{2,3}$=0.0098 $p^{1,3}$<0.0001	OR[1,2] 1.56 95% CI (1.38-1.77) OR[2,3] 0.93 95% CI (0.9-0.99) OR[1,3] 1.47 95% CI(1.3-1.67)
E	653 (52.37%)	12600 (49.46%)	5368 (38.2%)	p<0.0001 $p^{1,2}$=0.0485 $p^{2,3}$<0.0001 $p^{1,3}$<0.0001	OR[1,2] 1.12 95% CI (1.0-1.26) OR[2,3] 1.58 95% CI (1.52-1.65) OR[1,3] 1.77 95% CI (1.58-2.0)
F	94 (7.54%)	1868 (7.33%)	956 (6.8%)	p=0,1289	OR[1,2] 1.03 95% CI (0.82-1.28) OR[2,3] 1.08 95% CI (1.0-1.18) OR[1,3] 1.11 95% CI (0.89-1.39)
G	252 (20.21%)	3824 (15.01%)	2372 (16.88%)	p<0.0001 $p^{1,2}$<0.0001 $p^{2,3}$<0.0001 $p^{1,3}$=0.0032	OR[1,2] 1.43 95% CI (1.24-1.66) OR[2,3] 0.86 95% CI (0.82-0.92) OR[1,3] 1.24 95% CI (1.07-1.44)
H	714 (57.26%)	12875 (50.54%)	6531 (46.48%)	p<0.0001 $p^{1,2}$<0.0001 $p^{2,3}$<0.0001 $p^{1,3}$<0.0001	OR[1,2] 1.31 95% CI (1.17-1.47) OR[2,3] 1.17 95% CI (1.13-1.23) OR[1,3] 1.54 95% CI (1.37-1.74)
I	1247 (100.0%)	25474 (100.0%)	10548 (75.07%)	p<0.0001 $p^{1,2}$=1.0 $p^{2,3}$<0.0001 $p^{1,3}$<0.0001	OR[1,2] ∞ 95% CI (0-∞) OR[2,3] ∞ 95% CI (2291.37-∞) OR[1,3] ∞ 95% CI (112.01-∞)
J	1247 (100.0%)	14840 (58.26%)	14047 (99.97%)	p<0.0001 $p^{1,2}$<0.0001 $p^{2,3}$<0.0001 $p^{1,3}$=1.0	OR[1,2] ∞ 95% CI (241.79-∞) OR[2,3] 0.00 95% CI (0.0-0.0) OR[1,3] ∞ 95% CI (0.06-∞)
K	819 (65.68%)	15769 (61.9%)	8724 (62.09%)	p = 0,0273 $p^{1,2}$=0.008 $p^{2,3}$=0.7238 $p^{1,3}$=0.0132	OR[1,2] 1.17 95% CI (1.04-1.33) OR[2,3] 0.99 95% CI (0.95-1.04) OR[1,3] 1.16 95% CI (1.03-1.32)

L	269 (21.57%)	4348 (17.07%)	2778 (19.77%)	p<0.0001<br>$p^{1,2}$<0.0001<br>$p^{2,3}$<0.0001<br>$p^{1,3}$=0.1365	OR^{1,2} 1.33 95% CI (1.16-1.54)<br>OR^{2,3} 0.83 95% CI (0.79-0.88)<br>OR^{1,3} 1.11 95% CI (0.97-1.29)
M	720 (57.74%)	13036 (51.17%)	7193 (51.19%)	p<0.0001<br>$p^{1,2}$<0.0001<br>$p^{2,3}$=0.9805<br>$p^{1,3}$<0.0001	OR^{1,2} 1.30 95% CI (1.16-1.47)<br>OR^{2,3} 0.99 95% CI (0.96-1.04)<br>OR^{1,3} 3 1.30 95% CI (1.16-1.47)
N	548 (43.95%)	10495 (41.2%)	4907 (34.92%)	p<0.0001<br>$p^{1,2}$=0.0583<br>$p^{2,3}$<0.0001<br>$p^{1,3}$<0.0001	OR^{1,2} 1.11 95% CI (1.0-1.26)<br>OR^{2,3} 1.30 95% CI (1.25-1.36)<br>OR^{1,3} 1.46 95% CI (1.3-1.64)
Q	17 (1.36%)	244 (0.96%)	291 (2.07%)	p<0.0001<br>$p^{1,2}$=0.2027<br>$p^{2,3}$<0.0001<br>$p^{1,3}$=0.0881	OR^{1,2} 1.42 95% CI (0.82-2.35)<br>OR^{2,3} 0.45 95% CI (0.38-0.54)<br>OR^{1,3} 0.65 95% CI (0.37-1.07)
S	237 (19.01%)	4957 (19.46%)	3218 (22.9%)	p<0.0001<br>$p^{1,2}$=0.72<br>$p^{2,3}$<0.0001<br>$p^{1,3}$=0.0018	OR^{1,2} 0.97 95% CI (0.84-1.12)<br>OR^{2,3} 0.81 95% CI (0.77-0.86)<br>OR^{1,3} 0.78 95% CI (0.68-0.92)
T	153 (12.27%)	2290 (8.99%)	1539 (10.95%)	p<0.0001<br>$p^{1,2}$=0.0001<br>$p^{2,3}$<0.0001<br>$p^{1,3}$=0.1696	OR^{1,2} 1.41 95% CI (1.18-1.69)<br>OR^{2,3} 0.80 95% CI (0.75-0.86)<br>OR^{1,3} 1.13 95% CI (0.95-1.36)

Note: A and B - Certain infectious and parasitic diseases. C - Neoplasms. D - Diseases of the blood and blood-forming organs and certain disorders involving the immune mechanism. E - Endocrine, nutritional and metabolic diseases. F - Mental and behavioural disorders. G - Diseases of the nervous system. H - Diseases of the eye and adnexa; Dis-eases of the ear and mastoid process. I - Diseases of the circulatory system. J - Diseases of the respiratory system. K - Diseases of the digestive system. L - Diseases of the skin and subcutaneous tissue. M - Diseases of the musculoskeletal system and connective tissue. N - Diseases of the genitourinary system. Q - Congenital malformations, deformations and chromosomal abnormalities. S, T - Injury, poisoning and certain other consequences of external causes.

* Superscripts [1,2,3] denote the numbers of the groups being compared

Supplemental table 2. Number of visits to medical care during the observation period with diseases from different ICD-10 groups

ICD-10 group (first character of code)	AF+COPD (n=1,247)	AF (n=25,474)	COPD (n=14,051)	Significance level, p*
	**1**	**2**	**3**	
A	0.09±0.75	0.04±0.44	0.09±1.35	p<0.001<br>$p^{1,2}$=0.0002<br>$p^{2,3}$<0.0001<br>$p^{1,3}$=0.839
B	0.22±1.07	0.15±0.89	0.31±1.64	p<0.001<br>$p^{1,2}$=0.0126<br>$p^{2,3}$<0.0001

				$p^{1,3}=0.0446$
C	2.1±6.46	1.72±6.56	1.83±7.08	$p<0.001$<br>$p^{1,2}=0.0413$<br>$p^{2,3}=0.1087$<br>$p^{1,3}=0.1869$
D	1.38±3.41	0.85±2.39	0.96±2.63	$p<0.0001$<br>$p^{1,2}<0.0001$<br>$p^{2,3}<0.0001$<br>$p^{1,3}<0.0001$
E	4.08±8.12	3.55±7.78	2.35±6.29	$p<0.0001$<br>$p^{1,2}=0.0188$<br>$p^{2,3}<0.0001$<br>$p^{1,3}<0.0001$
F	0.3±1.95	0.26±1.71	0.31±2.29	$p<0.001$<br>$p^{1,2}=0.4079$<br>$p^{2,3}=0.0069$<br>$p^{1,3}=0.8381$
G	0.49±1.92	0.37±1.79	0.48±2.04	$p<0.0001$<br>$p^{1,2}=0.021$<br>$p^{2,3}<0.0001$<br>$p^{1,3}=0.827$
H	2.67±4.68	2.1±3.95	1.78±3.65	$p<0.0001$<br>$p^{1,2}<0.0001$<br>$p^{2,3}<0.0001$<br>$p^{1,3}<0.0001$
I	24.04±16.98	19.44±15.36	9.83±12.3	$p<0.0001$<br>$p^{1,2}<0.0001$<br>$p^{2,3}<0.0001$<br>$p^{1,3}<0.0001$
J	10.97±11.86	2.39±4.81	10.72±11.5	$p<0.0001$<br>$p^{1,2}<0.0001$<br>$p^{2,3}<0.0001$<br>$p^{1,3}=0.4639$
K	3.13±4.58	2.92±4.41	3.13±4.73	$p<0.001$<br>$p^{1,2}=0.095$<br>$p^{2,3}<0.0001$<br>$p^{1,3}=0.9571$
L	0.44±1.23	0.31±0.94	0.4±1.21	$p<0.0001$<br>$p^{1,2}<0.0001$<br>$p^{2,3}<0.0001$<br>$p^{1,3}=0.2011$
M	2.7±5.17	2.19±4.15	2.37±4.68	$p<0.0001$<br>$p^{1,2}<0.0001$<br>$p^{2,3}=0.0001$<br>$p^{1,3}=0.0187$
N	2.03±4.57	1.71±3.96	1.37±3.39	$p<0.0001$<br>$p^{1,2}=0.0071$<br>$p^{2,3}<0.0001$<br>$p^{1,3}<0.0001$
Q	0.03±0.39	0.03±0.64	0.05±0.59	$p<0.001$<br>$p^{1,2}=0.9059$<br>$p^{2,3}=0.0022$

				$p^{1,3}$=0.19
S	0.37±1.0	0.38±1.02	0.49±1.27	p<0.001<br>$p^{1,2}$=0.828<br>$p^{2,3}$<0.0001<br>$p^{1,3}$=0.0011
T	0.25±1.29	0.16±0.84	0.21±1.09	p<0.001<br>$p^{1,2}$=0.0008<br>$p^{2,3}$<0.0001<br>$p^{1,3}$=0.2796

Note: A and B - Certain infectious and parasitic diseases. C - Neoplasms. D - Diseases of the blood and blood-forming organs and certain disorders involving the immune mechanism. E - Endocrine, nutritional and metabolic diseases. F - Mental and behavioural disorders. G - Diseases of the nervous system. H - Diseases of the eye and adnexa; Diseases of the ear and mastoid process. I - Diseases of the circulatory system. J - Diseases of the respiratory system. K - Diseases of the digestive system. L - Diseases of the skin and subcutaneous tissue. M - Diseases of the musculoskeletal system and connective tissue. N - Diseases of the genitourinary system. Q - Congenital malformations, deformations and chromosomal abnormalities. S, T - Injury, poisoning and certain other consequences of external causes.

* Superscripts ^{1,2,3} denote the numbers of the groups being compared
